# Young adults at risk of early work disability: who are they?

**DOI:** 10.1186/s12889-018-6095-0

**Published:** 2018-10-16

**Authors:** Vigdis Sveinsdottir, Hege Randi Eriksen, Valborg Baste, Jørn Hetland, Silje Endresen Reme

**Affiliations:** 1 NORCE Norwegian Research Centre, Bergen, Norway; 2 Department of Sport, Food and Natural Sciences, Western Norway University of Applied Sciences, Bergen, Norway; 3 Department of Psychosocial Science, University of Bergen, Bergen, Norway; 4 Department of Psychology, University of Oslo, Oslo, Norway

**Keywords:** Young adults, NEET, Mental health, Bullying, Vocational rehabilitation, Disability, Unemployment

## Abstract

**Background:**

Young adults that are not in education, training or employment represent a problem across European countries. While some are cases of temporary transitions or short-term inactivity, others represent a more vulnerable group at risk of early work disability. Early exclusion from the labor market represents long lives exposed to detrimental effects of unemployment on health and well-being, and constitutes an economic burden for society. There is need for more knowledge about young adults who are at risk of early work disability but have not yet reached the point of more permanent exclusion. This study aims to investigate social and health-related problems in a Norwegian sample of young adults at risk of early work disability, and their self-perceived causes of illness.

**Methods:**

Baseline data from participants in the SEED-trial (*N* = 96), a randomized controlled trial comparing individual placement and support to traditional vocational rehabilitation in young adults at risk of early work disability, were analyzed. Background, health behaviors, adverse social experiences, disability level, physical and mental health, social support, coping, and self-perceived causal attributions of illness were measured. Gender differences were analyzed using chi-square and t-tests.

**Results:**

Mean age was 24, and 68% were men. One third reported reading and writing difficulties, and 40% had less than high-school education. The majority had experienced bullying (66%) or violence (39%), and 53% reported hazardous alcohol use. Psychological distress was the most prevalent health problem (52%), and women generally had more physical and mental health problems than men. Self-perceived causal attributions of illness were mainly related to relational problems, followed by health behaviors, heredity/genetics, and external environmental factors.

**Conclusions:**

The study provides a deeper insight into a vulnerable group with substantial challenges related to adverse social experiences, psychological distress, and alcohol use, who emphasized relational problems as the main causal factor for their illness. Findings suggest a need for broader focus on psychological and social factors in vocational rehabilitation efforts targeting young adults at risk of early work disability. Furthermore, gender-specific approaches may be warranted and should be followed up in future studies.

**Trial registration:**

Clinicaltrials.gov: NCT02375074. Retrospectively registered December 3rd 2014.

**Electronic supplementary material:**

The online version of this article (10.1186/s12889-018-6095-0) contains supplementary material, which is available to authorized users.

## Background

Young people who are not in employment, education, or training (NEET) represent a problem across Europe [1], causing worries about the potentially detrimental effects of unemployment on health and well-being [2, 3], as well as the economic burden for the society [4]. It has been estimated that 14.2% of young adults aged 15–29 In Europe were NEET in 2016 [5]. NEETs are a heterogeneous population, and while some are in between activities or short-term unemployed, others represent a more vulnerable group of individuals who have given up efforts in education and employment or remain unemployed for prolonged periods of time. The main risk factors for NEET status include poor self-perceived health, but non-medical factors such as low educational attainment and immigrant status have an even stronger impact [4]. Family background factors such as having parents who have little education or have experienced unemployment, further increase the likelihood of becoming NEET [4]. Uncertain or misaligned employment aspirations are also associated with future NEET status, especially among young men with low socioeconomic status, leading to broken transition phases for youth in a changing and increasingly individualized labor market [6]. Being outside education or employment can have significant ramifications on later participation and attachment to working life, and NEET status in early adulthood is associated with a clear and long-lasting risk of future social exclusion, including work disability [7].

The Nordic countries are generally characterized by low unemployment rates [8], and are among the countries with the fewest young adults having NEET status. While Norway has a relatively low NEET proportion of 7%, approximately half of Norwegian NEETs receive health-related benefits, and one in five remain in the same situation 5 years later [9]. The number of young adults aged 18–29 receiving permanent disability benefits in Norway has more than doubled during the last 10 years [10], while the population in the same age group has increased by 20% [11]. Risk factors for permanent disability benefits among young adults in Norway are similar to those of NEET status, and mainly concern socioeconomic factors such as lower education or income, poor social and family relations, and a weak connection to working life [12, 13]. Qualitative research on young disability recipients in Norway has furthermore underlined the importance of non-medical factors involving difficult childhoods, adjustment problems, and adverse social experiences related to abuse and bullying [14], although this remains to be investigated in larger follow-up studies.

Mental and behavioral disorders are among the leading causes for years lost to disability among youth in high-income countries [15], and are also the major reasons for early work disability in Norway, constituting the main diagnosis in 63% of cases [16]. Data from Norwegian registries show that while the increase in work disability among those aged 18–19 is mainly due to various intellectual and congenital disorders, the increase among those aged 20–29 is mainly due to other mental illness, including schizophrenia, pervasive developmental disorders, behavioral and personality disorders, followed by affective and anxiety disorders [17]. The gender distribution among young disabled contrasts with that of disability beneficiaries in the remaining population, with the majority (56%) being young men.

The Norwegian Labor and Welfare Administration provides work assessment allowance (WAA) for individuals with impaired working capacity who are unemployed or have exceeded the maximum duration of 1 year on sickness leave [18]. WAA is a temporary benefit that can be received for a maximum of 4 years, and if the earning capacity remains impaired, the next step may be to apply for permanent disability benefits. While receiving WAA, the individual is required to keep up to an activity plan involving ongoing treatment or participation in various employment schemes, while his or her work ability is being assessed. While employment schemes that focus on ordinary employment have gained international popularity [19], *traineeships in sheltered businesses* is a Norwegian employment scheme that is only used in cases of particularly uncertain professional abilities and impaired work capacity, that requires close and broad supervision and assistance [20]. While the impaired work capacity may be primarily caused by illness, it may in other cases be primarily due to social problems [21]. Young adults who are receiving temporary benefits and considered eligible for participation in sheltered traineeships represent a specifically challenged group of NEETs, at risk of early work disability and exclusion from working life at an early age.

Early exclusion from working life is subject to considerable societal interest and attention in Norway as well as other European countries, but there is little knowledge about the individuals who are at high risk but have not yet reached the more permanent point of disability benefits. There is need for further investigation to provide insight into who this group is in terms of social and health-related problems, and what they believe may have caused their illness.

### Aim

The aim of the study was to investigate the prevalence and level of various social and health-related problems and health behaviors in young adults at risk of early work disability in Norway, and to analyze possible gender differences. A secondary aim was to investigate to which factors participants who perceive themselves to have an illness attribute the cause of their illness.

## Methods

### Data and design

This study is based on baseline survey data on social and health-related variables from the randomized controlled trial “Supported Employment and preventing Early Disability” (the SEED-trial) [22]. The SEED-trial is an ongoing randomized controlled trial investigating the effect of individual placement and support vs. traditional vocational rehabilitation in individuals at risk of early work disability in Norway. For additional information about the trial, study design and procedures, see Sveinsdottir et al. 2016 [22].

### Participants and recruitment

**Fig. 1:**
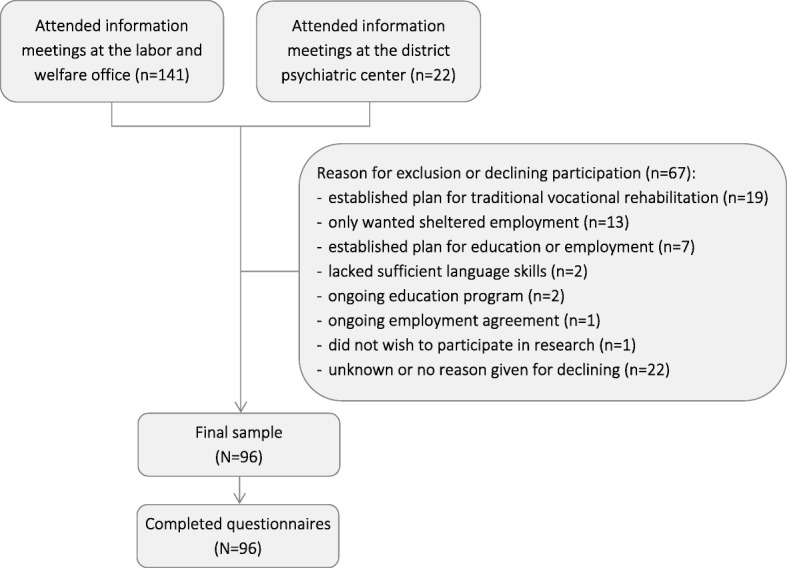
Flowchart of recruitment of participants

Ninety-six individuals (65 men (68%) and 31 women (32%)) with a mean age of 24 (SD = 3.25), participated in the study. Participants were young adults aged 18–29 in the year of inclusion, were not in employment or undergoing education, were receiving temporary benefits (mainly WWA, or employment scheme benefits), and were considered by their caseworkers at the Norwegian labor and welfare administration to be eligible for traineeships in sheltered businesses. Traineeships in sheltered businesses are only offered to those with impairment and particularly uncertain work capabilities requiring close follow-up. During June 2014 through December 2016, new eligible clients at one central and nine local labor and welfare offices in and around the city of Bergen, Norway, were referred to meetings to receive information about the study. Referrals were also made by a secondary care district psychiatric center in Bergen, with subsequent follow-up at the local labor and welfare office. At the information meetings, eligible participants were screened on two additional inclusion criteria, before being invited to participate in the trial: 1) Sufficient language skills to answer questionnaires in Norwegian, and 2) interest in receiving help to obtain ordinary work. There were no exclusion criteria based on diagnosis, and participants with any type of social and/or health-related problems were invited. A total of 163 participants attended the information meetings, whereof 67 were excluded or declined participation (Fig. 1).

### Data collection

Questionnaires were administered to all participants at the information meetings, either electronically or in paper format. Participants received help and assistance to answer the questionnaires upon request.

Electronic responses were collected using iPads with secure survey software (Qualtrics, Provo, UT), and stored in a secure online database. Responses in paper format were stored in a locked filing cabinet. Personal information and contact details were stored separately from the collected data, in a locked and fireproof safe.

### Questionnaire and instruments

In the first part of the questionnaire, information on self-reported background and social variables were collected. Dichotomous variables were computed for education level (less than high school vs. other), reading/writing difficulties (yes vs. no), marital status (single vs. other), living arrangements (living with parents vs. other), number of children (none vs. other), immigrant background (immigrants and Norwegian-born to immigrant parents vs. not), previous participation in employment scheme (yes vs. no), previous employment (yes vs. no), and reasons for unemployment (psychological problems vs. not, other health problems vs. not, and other non-health-related reasons vs. not). Participants were also asked to list whether they had received treatment during the last 6 months, and whether they had received consultations by general practitioners, psychiatrists, psychologists, physio−/manual therapists, chiropractors, or other therapists. Dichotomization of continuous background variables was based on visual inspection of the distributions.

#### Disability level

Disability level was measured using the 12-item self-administered version of the WHO Disability Assessment Schedule 2.0 (WHODAS 2.0), consisting of a sum-score (Cronbach’s α = .87) based on six domains of life: Cognition, mobility, self-care, getting along, life activities, and participation [23]. Each item was scored on a 5-point scale ranging from 0 (none) to 4 (extreme or cannot do). A simple scoring strategy without weighting of individual items was used, ranging from 0 (no disability) to 48 (full disability). In cases of up to five missing items, the mean of the remaining items was calculated and multiplied by 12. In addition to the sum-score, cut-off scores of ≥10 for significant disability were used based on the top 10% of the population in normative data [24].

#### Health behaviors: Alcohol and drug use

Alcohol use was measured using the 3-item Alcohol Use Disorders Identification Test (AUDIT-C) [25]. Items were scored on 5-point scales ranging from 0 to 4, with higher scores indicating higher frequency and quantity of alcohol consumption. Based on a sum-score, validated cutoff-scores of ≥4 for men and ≥ 3 for women were used to indicate hazardous drinking or active alcohol use disorders [26].

Drug use was measured using the 11-item Drug Use Disorders Identification Test (DUDIT) [27]. Items 1–9 were scored on 5-point scales ranging from 0 to 4, and items 10–11 were scored on 3-point scales as 0, 2, and 4, with higher scores indicating more severe drug use. Based on a sum-score, validated cutoff-scores of ≥6 for men and ≥ 2 for women were used to indicate drug-related problems [28].

#### Bullying

A new eight-item questionnaire was developed in collaboration with Dan Olweus and Jørn Hetland, researchers within the fields of bullying in school and bullying in working life, respectively. The new measure was specifically developed in order to measure lifetime experiences with bullying victimization and bullying perpetration in different social arenas, for respondents who are currently not in employment, education, or training. The items were preceded by a description of bullying according to Olweus’s definition [29], describing that bullying can be direct and indirect as well as verbal and physical, and that it involves a perceived power imbalance or difficulty defending oneself. It was emphasized that friendly teasing, and fights or arguments between equal parts were not regarded as bullying.

Bullying victimization was measured using five single items, concerning three arenas: School (2 items, bullied by other students or teachers), working life (2 items, bullied by colleagues or leaders), and other social arenas (1 item). The items were scored on a 5-point scale ranging from 0 (never or almost never), 1 (one short period (a few weeks)), 2 (several shorter periods), 3 (one long period (several months)), to 4 (several longer periods of my time in school/working life/other social arenas). Values ≥2 within each arena were coded as bullying in that arena, and an overall dichotomous variable was created for bullying victimization in any arena vs. bullying in no arena. This is in line with the Olweus definition emphasizing repeated incidences over time rather than the length of an incidence [29], and coincides with how bullying has been categorized in other studies [30].

Bullying perpetration was measured with three single items, corresponding to the method and scale used for bullying victimization as described above. The questions concerned whether the participant him/herself had bullied others in three arenas: School (1 item), working life (1 item), and other social arenas (1 item). Values ≥2 within each arena were coded as bullying in that arena, and an overall dichotomous variable was created for bullying perpetration in any arena vs. bullying in no arena.

An additional dichotomous variable was created for those who reported that they were both victims and perpetrators of bullying (bully-victims).

#### Violence

Violence was measured using a single item concerning whether participants had been the victim of violence inflicted by others (not counting accidents and common children’s fights). If yes, participants were further asked to indicate what types of violent acts they had experienced (being hit, robbery/assault, sexual violence, deprivation of liberty, severe threats, or other), and whether incidents were single or repeated.

#### Psychological distress

Psychological distress was measured using the 25-item Hopkins Symptom Checklist (HSCL-25), consisting of two subscales: An anxiety dimension (10-items, α = .82) and a depression dimension (15-items, α = .91), in addition to a mean score (α = .93) [31]. Each item was scored on a 4-point scale ranging from 1 (no symptoms) to 4 (severe symptoms). In addition to the mean score, a validated cut-off score of ≥1.75 was used for psychological distress [32, 33].

#### Fatigue

Fatigue was measured using the 11-item Chalder Fatigue Questionnaire (CFQ) consisting of two subscales: Physical fatigue (7 items, α = .88) and mental fatigue (4 items, α = .67), in addition to a sum-score (α = .86) [34]. Each item was scored on a 4-point scale ranging from 0 (less than usual) to 3 (much worse than usual). In addition to the sum-score, a binary global fatigue score ranging from 0 to 11 was calculated and validated cut-off scores of ≥4 were used for severe fatigue [34, 35].

#### Sleep problems (insomnia)

Three single items were developed in collaboration with Mari Hysing, researcher within the field of mental health and sleep problems in children and adolescence, to serve as a simple proxy for the diagnostic criteria for insomnia according to the Diagnostic and Statistical Manual of Mental Disorders, fifth edition (DSM-5) [36]. The first item concerned problems with falling asleep, waking up at night, and/or waking up too early. Respondents were asked to indicate how many nights they experienced each problem during a typical week, on a scale ranging from 0 to 7. If any sleep problems were reported, respondents were asked to proceed to the second and third items, indicating how long the problems had lasted, and how many times a week the problems affected daily life. A dichotomous variable for insomnia was computed based on whether or not one or more sleep problems exceeded three nights a week, had lasted more than 3 months, and affected daily life for more than 3 days a week.

#### Subjective health complaints

Subjective health complaints were measured using the 29-item Subjective Health Complaints Inventory (SHC), consisting of five subscales: Musculoskeletal pain (8 items, α = .78), pseudoneurology (7 items, α = .73), gastrointestinal problems (7 items, α = .64), allergy (5 items, α = .48), and flu (2 items, α = .56), in addition to a sum-score (α = .82) [37]. Each item was scored on a 4-point scale ranging from 0 (no complaints) to 3 (serious complaints).

#### Global well-being

Global well-being was measured using a 10-point Cantril Ladder Scale [38], ranging from 1 (the worst life possible) to 10 (the best life possible), asking respondents to indicate on which step of the ladder they feel they stand today, on which step they would say they stood a year ago, and where they believe they will be a year from now.

#### Social support

Social support was measured using 11-items of the Nondirective and Directive Support Survey [39] as suggested by Øyeflaten et al. (2010), using two subscales: Directive social support (4 items, α = .73) and nondirective social support (7 items, α = .84) [40]. The directive subscale involves instructive support and taking charge of the situation in order to help the recipient, while nondirective support is of a more cooperative nature and involves acceptance of the recipients own thoughts and choices [40]. Each item was scored on a 5-point scale ranging from 1 (not at all typical) to 5 (very typical). The survey also instructs respondents to indicate the specific person to whom they turn for support, and whether this is their doctor, spouse/partner, or “other” including an open response. An additional dichotomous variable was created based on a categorization of whether the support provider was a professional (e.g. doctor, psychologist) vs. personal (e.g. partner, family, friend).

#### Coping

Coping was measured using the 7-item Theoretically Originated Measure of the Cognitive Activation Theory of Stress (TOMCATS) [41], consisting of three subscales: Coping (1 item), helplessness (3 items, α = .65), and hopelessness (3 items, α = .66). Each item was scored on a 4-point scale ranging from 1 (completely true) to 4 (not true at all). Items were reversed in order for higher scores to reflect higher degrees of coping, helplessness, or hopelessness. Mean scores were calculated for the helplessness and hopelessness subscales.

#### Illness perceptions

Illness perceptions were measured using the 9-item Brief Illness Perception Questionnaire (BIPQ), where each item measures a different dimension of illness perceptions: *Consequences*—how much the illness affect your life; *timeline*—how long you believe the illness will last; *personal control*—how much control you feel over the illness; *treatment control*—how much you think treatment can help the illness; *identity*—how much you experience symptoms from the illness; *concern*—how concerned you are about the illness; *coherence*—how well you understand the illness; *emotional response*—how much the illness affects you emotionally; and a causal attribution item [42]. Items 1–8 were scored on 11-point scales ranging from 0 to 10, with higher scores reflecting an increasingly threatening view of the illness. Item 9 was open-ended and concerned causal attribution: “Please list in rank-order the three most important factors that you believe caused your illness”. Participants who did not perceive themselves as having any illness, were told to skip this questionnaire.

### Data analyses

Descriptive statistics were calculated for the total sample, and by gender. Gender differences were analyzed by chi-square tests for the dichotomous variables and independent t-tests for the continuous variables.

Responses to the open-ended item in the BIPQ regarding causal attribution were categorized using thematic analysis, as described by Joffe & Yardley [43]. Themes were identified and data was categorized into coding categories using a descriptive and inductive approach. A coding manual including category definitions was prepared (Additional file 1), and categorization was performed independently by two authors to determine inter-rater reliability. In cases of inconsistency, categorization was discussed until consensus was reached.

In order to maintain the anonymity of respondents, values with fewer than five respondents are not reported.

Statistical analyses were performed using IBM SPSS Statistics, Versions 24.0 and 25.0. The significance level was set to α = .05.

## Results

### Background, alcohol and drug use

The majority of participants were male, single, childless, and nearly half were living with their parent(s). Forty percent had less education than high-school and 33% reported reading or writing difficulties (see Table 1 for more background information). Fifty-three percent of participants reported hazardous drinking or active alcohol use disorders, while 15% reported any drug use and 10% scored above the cut-off for drug-related problems. Men were more often single than women and more often reported non-health-related reasons for unemployment.

**Table 1:** Background, alcohol and drug use. Total score and comparison of genders

	Total (*N* = 96)		Men (*n* = 65)		Women (*n* = 31)		*p*-value
	n	%	n	%	n	%	
Education							
Less than high school	38	(40)	28	(43)	10	(32)	.311
Reading/writing difficulties	32	(33)	24	(37)	8	(26)	.280
Marital status							
Single	68	(71)	53	(82)	15	(48)	**< .001**
Living arrangements							
With parent(s)	44	(46)	32	(49)	12	(39)	.333
Children							
None	62	(86)	41	(87)	21	(84)	.730^a^
Country of birth							
Immigrant background	15	(17)	10	(16)	5	(17)	1.000^a^
Employment							
Previous employment scheme	59	(64)	41	(67)	18	(58)	.387
Previous employment	56	(59)	35	(55)	21	(68)	.225
Reason for unemployment							
Psychological problems	51	(53)	32	(49)	19	(61)	.268
Other health problems	33	(34)	23	(35)	10	(32)	.763
Other, non-health-related	32	(33)	26	(40)	6	(19)	**.045**
Alcohol use							
Over gender cutoff	51	(53)	33	(51)	18	(58)	.503
Drug use							
Any drug use	14	(15)	9	(14)	5	(16)	.765^a^
Over gender cutoff^b^	10	(10)	–	–	–	–	–

^a^1 cell had an expected cell count less than 5. Exact *p* value (Fisher’s exact test significance) was used

^b^ Values for groups with fewer than five respondents in either group are not reported

All values in boldface in the p-value column are statistically significant at the 0.05 or 0.001 level

### Adverse social experiences

Among the participants, 66% reported that they had experienced bullying, with school being the most common arena. Fourteen percent reported having bullied others, and 8% were both victims and perpetrators of bullying. A total of 39% of participants reported that they had experienced violence, of which 67% reported repeated incidences. Being hit was the most common type of violence for both genders, and 29% of women reported sexual abuse. There were no gender differences in exposure to either bullying or violence (Table 2).

**Table 2:** Adverse social experiences, total score and comparison of genders

	Total (*N* = 96)		Men (*n* = 65)		Women (*n* = 31)		*p*-value
	n	%	n	%	n	%	
Bullying, victim	63	(66)	41	(63)	22	(71)	.447
School	56	(58)	36	(55)	20	(65)	
Work^a^	9	(16)^b^	–	–	–	–	
Other social arenas^a^	23	(24)	–	–	–	–	
Bullying, perpetrator^a^	13	(14)	–	–	–	–	
Bullying, victim and perpetrator^a^	8	(8)	8	(12)	–	–	
Violence	37	(39)	23	(35)	14	(45)	.357
Been hit	27	(28)	17	(26)	10	(32)	
Severe threats	14	(15)	9	(14)	5	(16)	
Sexual abuse/violence^a^	–	–	–	–	9	(29)	
All other	15	(16)	9	(14)	6	(19)	
Violence, repeated incidences	24	(67)^c^	13	(59)	11	(79)	

^a^ Values for groups with fewer than five respondents are not reported

^b^ Percentage of those who had previously worked (*n* = 56)

^c^ Percentage of those who had experienced violence and reported frequency (*n* = 36)

### Health, coping and social support

With the exception of gastrointestinal complaints and global well-being, women consistently reported more physical and mental health problems than men (Table 3). Men also reported higher levels of coping, while women received more nondirective social support than men.

**Table 3:** Health, coping, and social support. Total score and comparison of genders

	Total (*N* = 96)	Men (*n* = 65)	Women (*n* = 31)	*p*-value
	Mean ± SD	Mean ± SD	Mean ± SD	
Disability, 0–48	8.60 ± 7.54	7.56 ± 6.98	10.80 ± 8.29	**.048**
Psychological distress, 1–4	1.85 ± 0.55	1.74 ± 0.47	2.09 ± 0.63	**.007**
Depression, 1–4	1.95 ± 0.64	1.84 ± 0.58	2.18 ± 0.73	**.014**
Anxiety, 1–4	1.70 ± 0.49	1.58 ± 0.41	1.96 ± 0.55	**.001**
Fatigue, 0–33	13.43 ± 5.82	12.41 ± 5.28	15.64 ± 6.40	**.011**
Physical, 0–21	8.68 ± 4.40	8.06 ± 4.22	10.01 ± 4.57	**.045**
Psychological, 0–12	4.75 ± 2.22	4.34 ± 1.90	5.63 ± 2.62	**.019**
Subjective health complaints, 0–87	14.57 ± 9.54	12.02 ± 7.28	19.75 ± 11.45	**.001**
Musculoskeletal, 0–24	4.16 ± 3.95	3.20 ± 3.21	6.12 ± 4.59	**.003**
Pseudoneurology, 0–21	5.76 ± 3.93	4.99 ± 3.43	7.35 ± 4.46	**.012**
Gastrointestinal, 0–21	2.40 ± 2.87	1.91 ± 2.07	3.37 ± 3.90	.059
Global well-being, 1–10				
Today	4.85 ± 1.79	4.79 ± 1.66	5.00 ± 2.07	.593
Past (1 year)	4.00 ± 2.23	4.05 ± 2.05	3.90 ± 2.59	.767
Future (1 year)	7.02 ± 2.13	6.93 ± 2.26	7.22 ± 1.85	.544
Social support				
Nondirective support, 1–5	3.87 ± 0.85	3.70 ± 0.87	4.23 ± 0.67	**.004**
Directive support, 1–5	3.09 ± 0.95	3.10 ± 0.96	3.07 ± 0.94	.874
Coping				
Coping, 1–4	2.63 ± 0.72	2.77 ± 0.68	2.33 ± 0.71	**.006**
Helplessness, 1–4	2.34 ± 0.69	2.33 ± 0.70	2.36 ± 0.70	.857
Hopelessness, 1–4	2.28 ± 0.74	2.21 ± 0.69	2.44 ± 0.82	.176

All values in boldface in the p-value column are statistically significant at the 0.05 or 0.01 level

According to predefined cut-off values, 52% of participants reported psychological distress, 42% had severe fatigue, and 32% reported severe disability. Twenty-eight percent of participants reported sleep problems corresponding to the DSM-5 criteria for insomnia [36].

Most participants had received treatment during the last 6 months (79%), mainly by their general practitioner (56% of all participants) or by a psychologist/psychiatrist (50% of all participants), while 10% of participants had received treatment by physiotherapist/manual therapist and/or chiropractor, and 14% of participants reported receiving other treatment. More women reported receiving treatment than men (*p* = .017), which was mainly explained by more women receiving treatment by psychologists and/or psychiatrists (*p* = .016).

### Illness perceptions and causal attributions

Only participants who perceived themselves as having an illness were told to fill out the BIPQ, and a total of 72 participants (75%) responded (Table 4). Women had a higher belief in treatment being helpful for their illness, and reported that they worried more about their symptoms, than men.

**Table 4:** Illness perceptions, total score and comparison of genders; and causal attributions, response categories and examples

	Total (*N* = 72)	Men (*n* = 48)	Women (*n* = 24)	
Continuous items, 0–10	Mean ± SD	Mean ± SD	Mean ± SD	*p*-value
Consequences	5.85 ± 3.08	5.48 ± 2.97	6.58 ± 3.22	.153
Timeline	7.38 ± 3.21	7.69 ± 3.15	6.78 ± 3.33	.275
Personal control	5.69 ± 3.18	5.36 ± 3.17	6.33 ± 3.17	.226
Treatment control	4.28 ± 2.98	4.88 ± 2.95	3.21 ± 2.78	**.026**
Identity	5.66 ± 2.81	5.20 ± 2.70	6.50 ± 2.87	.069
Concern	4.63 ± 3.01	4.11 ± 2.78	5.63 ± 3.24	**.045**
Coherence	3.82 ± 3.23	3.45 ± 3.16	4.54 ± 3.30	.178
Emotional response	6.18 ± 3.09	6.09 ± 2.96	6.38 ± 3.39	.711
Causal attribution, open-ended	Number of responses^a^	Example of response		
Relational	20	“Loneliness”		
Health behavior	16	“Used various types of drugs”		
Hereditary/genetic	13	“Genetics”		
External environment	11	“Living situation”		
Bullying	6	“Bullied in childhood”		
Childhood	6	“A lot of moving [...] during my first 7 years”		
Psychological	6	“Social anxiety”		
Self-control/coping	5	“Bad choices”		
Traumatic life events	5	“Sexual abuse”		
Unknown	5	“Cause not explained”		
Other categories^b^	18			

^a^51 participants provided 111 open-ended responses

^b^Categories with fewer than five respondents (somatic, injury, pressure/demands, financial, fate/fortune) are not reported

All values in boldface in the p-value column are statistically significant at the 0.05 level

Among those who perceived themselves to have an illness, 51 participants (71%) provided a total of 111 different open-ended responses to the causal attribution item. The most common categories were relational problems, followed by health behaviors, heredity/genetics, and external environmental factors (Table 4). Inter-rater reliability for the categorization, as measured by Cohen’s Kappa, was high (κ = .91).

## Discussion

Main findings showed a group of NEETs at risk of early work disability, with substantial challenges related to adverse social experiences. Participants also reported high levels of psychological distress and alcohol use, and emphasized relational problems as the main causal factor when asked about their illness perceptions. Women generally reported more physical and mental health problems than men, while men more often reported non-health-related reasons for unemployment.

The low educational attainment found among participants is in line with major risk factors for NEET status and early work disability [4, 12]. Correspondingly, levels of reading and writing difficulties were approximately four times higher than that of a representative sample of Norwegian adolescents [44]. Furthermore, the rate of participants reporting hazardous drinking or active alcohol use disorders appears exceedingly high. It is however comparable to that of Norwegian college and university students [45], indicating that the level of consumption is not specific to the group of NEETs at risk of early work disability, but may rather indicate a problem on a societal level. Meanwhile, it could be argued that high levels of alcohol consumption may represent a more worrying problem when observed in a population not involved in employment or educational activities. Being unemployed after leaving school is associated with higher risk-related behavior, including substance abuse and dependence [46]. This coincides with the findings from the current study, as drug use was five times more prevalent than what has been found in normative data [28].

The findings of adverse social experiences in this group were considerable. Among those who had experienced violence, the majority reported repeated incidences, and a large proportion of female participants had been victims of sexual violence. Two in three participants reported being bullied in their past. Levels of bullying are difficult to compare directly to other studies, due to participants being outside of education and employment, which rendered existing measures inappropriate in this setting. However, although the measure used in the current study is broader than previous that of conventional studies of recent bullying in school, the level still appears substantial as compared to a prevalence of 8% in the Norwegian school population [47]. Victimization by bullying has been associated with a range of physical and psychological health problems, relational problems, and lower educational achievement [48]. It can be said to throw a long shadow across the lives of its victims [48], having long-lasting detrimental effects on the individual. Although issues of direction and causality remain unclear, longitudinal designs controlling for pre-existing risk-factors such as earlier symptoms suggest that victims of school bullies have a higher prevalence of psychotic experiences in later adolescence [49], and are at higher risk of depression up to 36 years later [50]. The proportion of bullying perpetration also appeared large, as 14% reported having bullied others, while 3–4% in the general population agree to have bullied others in school [47]. The antisocial behavior of bullying perpetration has previously been associated with negative childhood factors such as high levels of disruptive behavior disorders and social/family hardships [51], and is a strong predictor for future criminality [52]. Furthermore, 8% of participants reported the combination of both bullying victimization and perpetration, a group referred to as bully-victims. Bully-victims have been associated with poorer social and emotional adjustment as seen in victims, in addition to the problem behaviors associated with perpetration, and may represent an especially high-risk group [53].

Levels of coping were lower than that seen in a healthy Norwegian working population [54], which may be expected in a marginalized group at risk of early work disability. The finding that men experienced higher coping than women is however interesting. This can be seen in combination with the findings that women reported worrying more about their symptoms, had a higher belief in treatment being helpful for their illness, and more often sought treatment than men. Patterns of social support also differed between genders, as women reported more nondirective social support than men, and more often had a partner. Comparable studies on NEETs concerning coping and worrying are scarce, but the literature in general suggests that women may be more prone to rumination over symptoms and distress than men, which may contribute in explaining the greater rates of depression among women [55]. Higher rates of treatment seeking among women have been shown in previous studies of e.g. depression [56, 57] and generalized anxiety [58], and genders are likely to differ in how illness perceptions influence coping strategies such as seeking treatment and social support [59]. When asked to indicate one or more reasons for their unemployment, most participants stated psychological problems to be the main cause, while other health-problems and non-health-related problems were equally common. Men did however more often report non-health-related reasons than women, while women experienced more physical and mental health problems.

While NEET status may be a result of poor health, being NEET can also have severe individual consequences on mental and physical health [4]. About one in three had significant levels of disability as opposed to one in ten in normative data [24], and participants displayed high levels of various mental health symptoms including substantial psychological distress and fatigue compared to the general population [32, 60]. Accordingly, severity of pseudoneurological complaints such as tiredness, sadness and anxiety was also high, while musculoskeletal and gastrointestinal complaints were comparable to that of the general population aged younger than 30 [61]. The findings correspond to the diagnoses of young adults that are already receiving disability benefits in Norway, in which mental health problems is the major contributor [16].

The importance of psychological distress and the high prevalence of bullying in this group was further illustrated by participants’ self-perceived causal attributions of illness, which mainly concerned different psychosocial factors. The most common causal attribution was relational problems, which included repeated accounts of loneliness, isolation, lack of adequate care, or loss of love or friendship. Additional attributions were directly made to bullying, childhood, and traumatic life events. Few attributions were made to somatic problems or injuries. The exception were hereditary or genetic causal factors, which coincides with register studies showing that intellectual and congenital disorders are common among the youngest group of people with work disability in Norway [17].

While global well-being is generally high in the Norwegian population [62], participants rated their global well-being below the center of the ten-point scale. Albeit low, participants estimated that their level of well-being had been poorer 1 year earlier, and expectations about the future revealed a certain optimism among the participants, as participants on average predicted an increase to 7 in the next year which is closer to the Norwegian population mean at 7.8.

### Implications

Given the heterogeneity of the NEET population in Europe, there is need for research and policy measures to target specific subgroups [1, 63], and the current study focuses on a particularly vulnerable group of NEETs at risk of early work disability in Norway. The findings of health-related challenges within the group of young adults in this study are not unexpected when considering the inclusion criteria; all participants were NEETs receiving temporary benefits related to impaired working capacity and were considered to need special assistance with close follow-up. While levels of psychological distress were high, they correspond to previous knowledge about the major reasons for early work disability in Norway [16]. The more notable findings with important implications for measures targeting this group are related to psychosocial factors, including the high prevalence of bullying and exposure to violence. Even though only individuals who considered themselves to have an illness were told to respond to questions related to self-perceived causal attributions, the most common responses were related to non-medical causes, especially relational problems such as loneliness and isolation. In addition to preventive measures to reduce social exclusion by bullying [64] and early dropout [65], the findings call for a broader focus on social as well as psychological factors in vocational rehabilitation efforts for NEETs at risk of early work disability. Furthermore, the needs of women versus men may vary and cause need for gender-specific tailoring in vocational and treatment approaches.

### Strengths and limitations

Due to the low number of participants, and multiple comparisons of the large number of outcome measures included in the study, results from analyses comparing gender should be interpreted with caution.

Participants who did not perceive themselves to have an illness were told not to answer the BIPQ, which was reflected in the response rate to this questionnaire (75%). The Norwegian translation of the BIPQ translates illness to a term which may insufficiently emphasize the subjective feeling of illness and may be interpreted as “disease”. The distinction between disease, illness, and sickness found in the English language is less defined in Norwegian, which may have led participants who did not perceive their illness as an objectively defined disease to not respond. The finding that one in three participants also answered “non-health-related” when asked about their reasons for unemployment, does however indicate that participants may indeed have not responded to the BIPQ because they perceived their problems to be unrelated to their health.

The broad invitation to participate in the information meetings in the current study included everyone in the target group attending the majority of local labor and welfare offices in the second-largest city in Norway. However, the total number of invitations issued is unfortunately not known. Attrition due to missing invitations or invited participants not attending the information meetings can therefore not be determined. While it is possible that more vulnerable individuals may have missed invitations or declined participation in some cases, several declinations or exclusions of participants were due to ongoing or established plans for employment or education, indicating that the opposite may be true in other cases. Based on these considerations, combined with the long recruitment period and complete response rate among participants, we believe that the sample represents an important segment of young adults at risk of early work disability in Norway, namely those who are on the path towards permanent disability, but who still have a hope of gaining employment.

## Conclusions

The results of this study provide a deeper insight into a vulnerable group of NEETs who are at risk of early work disability. Findings of substantial challenges related to bullying, psychological distress, and alcohol use, combined with participants’ own causal attributions of illness, emphasize the importance of psychological and relational factors in vocational rehabilitation efforts targeting this important and marginalized group, who are at risk of being permanently excluded from the labor market at an early age. Furthermore, gender-specific approaches may be warranted, and should be followed up in future studies.

## Additional file

Additional file 1: Coding manual for self-perceived causal attributions of illness (open responses to the Brief Illness Perception Questionnaire). (PDF 16 kb)

## Abbreviations

BIPQ: Brief illness perception questionnaire
DSM-5: Diagnostic and statistical manual of mental disorders, fifth edition
NEET: Young people that are not in employment, education, or training
SD: Standard Deviation
SEED-trial: Supported employment and preventing early disability
WAA: Work assessment allowance
