# Supplementary material for: Young adults at risk of early work disability: who are they?
Source: BMC Public Health. 2018 Oct 16;18:1176. doi: 10.1186/s12889-018-6095-0 (PMC6192296; doi:10.1186/s12889-018-6095-0)
Supplement: Supplementary file 1 — Coding manual for self-perceived causal attributions of illness (open responses to the Brief Illness Perception Questionnaire). (PDF 16 kb) [file 12889_2018_6095_MOESM1_ESM.pdf]

Coding manual

Coding manual for self-perceived causal attributions of illness (open responses to the Brief Illness Perception Questionnaire).

1. Relational
   This category includes all responses focusing on relational problems, including loneliness and isolation, end of relationships or friendships, family problems, and lack of social support and care. This includes relational problems in childhood, but excludes bullying, pressure, and violence, which should be placed elsewhere.

2. Bullying
   This category involves all responses mentioning bullying in any arena as a causal factor.

3. Childhood
   Responses that emphasize upbringing and childhood, including living situation during childhood, are placed here. This does however exclude accounts of bullying, violence, and relational/family problems, although occurring in childhood, which should be placed in their respective categories.

4. Psychological
   Causal attributions to psychological complaints and symptoms of mental illness, such as anxiety and depression, should be placed here. This includes e.g. fear and insecurity, but excludes attributions to coping responses characterized as poor by the respondent.

5. Self-control/coping
   This category includes causal attributions to poor coping responses or lack in self-control, such as motivational problems and poor choices in life.

6. Health behavior
   Responses concerning substance abuse (both alcohol and drugs), sleep, diet, lifestyle, physical activity or inactivity, are placed here.

7. Somatic
   This category includes physiological health complaints, pain, and somatic factors that are attributed as the causal factor. Injuries caused by accidents are excluded.

8. Injury
   All references to accidents, e.g. fractures and physical trauma. Deliberately inflicted injury, e.g. violence, is however excluded.

9. Hereditary/genetic
   Responses stating genetic and hereditary causal factors are placed here. This includes congenital illness.

10. External environmental
    This category concerns external factors related to the society or environment that the person is living in. This includes references to society, institutions (e.g. penal or educational institutions), living conditions and general life situation.

11. Financial
    This category involves all responses concerning personal economy and financial problems.

12. Pressure/demands
    This category includes pressure, demands, and general stress, whether the source is external or internal, e. g. pressuring oneself too much.

13. Traumatic life events
    All traumatic life events belong in this category, such as accounts of abuse, violence, and rape. This also includes experiences where the nature of the event is not specified, but where an unnamed event is emphasized as the causal factor.

14. Fate/fortune
    Reponses concerning a predetermined course of events due to destiny or fate, along with attributions to uncontrollable factors determined by bad luck or fortune, are placed here.

15. Unknown
    This category concerns responses where causal factors are stated as none, or unknown, or not understood.
